# MoAIMS: efficient software for detection of enriched regions of MeRIP-Seq

**DOI:** 10.1186/s12859-020-3430-0

**Published:** 2020-03-14

**Authors:** Yiqian Zhang, Michiaki Hamada

**Affiliations:** 1 Department of Electrical Engineering and Bioscience, Faculty of Science and Engineering, Waseda University, 55N-06-10, 3-4-1 Okubo Shinjuku-ku, Tokyo, 169-8555 Japan; 2 AIST-Waseda University Computational Bio Big-Data Open Innovation Laboratory (CBBD-OIL), 3-4-1, Okubo Shinjuku-ku, Tokyo, 169-8555 Japan; 3 Artificial Intelligence Research Center, National Institute of Advanced Industrial Science and Technology (AIST), 2-41-6 Aomi, Koto-ku, Tokyo, 135-0064 Japan; 4 Institute for Medical-oriented Structural Biology, Waseda University, 2-2, Wakamatsu-cho Shinjuku-ku, Tokyo, 162-8480 Japan; 5 Graduate School of Medicine, Nippon Medical School, 1-1-5, Sendagi, Bunkyo-ku, Tokyo, 113-8602 Japan

**Keywords:** RNA modification, N6-methyladenosine, Negative binomial model, Treatment effect

## Abstract

**Background:**

Methylated RNA immunoprecipitation sequencing (MeRIP-Seq) is a popular sequencing method for studying RNA modifications and, in particular, for N6-methyladenosine (m6A), the most abundant RNA methylation modification found in various species. The detection of enriched regions is a main challenge of MeRIP-Seq analysis, however current tools either require a long time or do not fully utilize features of RNA sequencing such as strand information which could cause ambiguous calling. On the other hand, with more attention on the treatment experiments of MeRIP-Seq, biologists need intuitive evaluation on the treatment effect from comparison. Therefore, efficient and user-friendly software that can solve these tasks must be developed.

**Results:**

We developed a software named “model-based analysis and inference of MeRIP-Seq (MoAIMS)” to detect enriched regions of MeRIP-Seq and infer signal proportion based on a mixture negative-binomial model. MoAIMS is designed for transcriptome immunoprecipitation sequencing experiments; therefore, it is compatible with different RNA sequencing protocols. MoAIMS offers excellent processing speed and competitive performance when compared with other tools. When MoAIMS is applied to studies of m6A, the detected enriched regions contain known biological features of m6A. Furthermore, signal proportion inferred from MoAIMS for m6A treatment datasets (perturbation of m6A methyltransferases) showed a decreasing trend that is consistent with experimental observations, suggesting that the signal proportion can be used as an intuitive indicator of treatment effect.

**Conclusions:**

MoAIMS is efficient and easy-to-use software implemented in R. MoAIMS can not only detect enriched regions of MeRIP-Seq efficiently but also provide intuitive evaluation on treatment effect for MeRIP-Seq treatment datasets.

## Background

RNA modification, represented by the epitranscriptome [1], refers to biochemical modifications of RNAs that are involved in functional regulations such as translation efficiency and mRNA stability without a change in the RNA sequence. Over 100 types of RNA modifications have been reported [2]. Among them, researchers have recently focused on certain abundant modifications such as N6-methyladenosine (m6A) [3], N1-methyladenosine (m1A) [4], and 5-methylcytidine (m5C) [5].

With the fast growth of next-generation sequencing (NGS), scientists can study RNA modifications at a whole-transcriptome scale. Methylated RNA immunoprecipitation sequencing (MeRIP-Seq) is a type of NGS technology for studying RNA modifications and is particularly widely used to detect m6A, a modification found in various species including human, mouse, and zebrafish [6–8]. In MeRIP-Seq, an antibody specific to a certain type of RNA modification (such as m6A or m1A) is used to immunoprecipitate RNA; it is similar to another popular sequencing technology, i.e., ChIP-Seq (Chromatin immunoprecipitation sequencing) [9], which is used in studies of transcription factor binding. However, based on the inherent features of DNA and RNA, there is some difference between MeRIP-Seq and ChIP-Seq data. First, the distribution of ChIP-Seq read counts is relatively uniform while that of MeRIP-Seq is more variable owing to transcript abundance so that MeRIP-Seq requires an input RNA-Seq sample as a control. Second, transcript abundance affects the duplication rate, which must be considered in preprocessing MeRIP-Seq data. Third, because RNA sequencing can store strand information, which provides more accurate transcriptome profiling by strand-specific protocols [10], strand information must be well utilized when analyzing MeRIP-Seq data.

Commonly used tools for identifying enriched regions of MeRIP-Seq include MACS [11], exomePeak [12], and MeTPeak [13]. MACS, which is a popular software in ChIP-Seq analysis, assumes the Poisson distribution for read counts. Applying MACS in MeRIP-Seq analysis requires the genome size to be set [14]; furthermore, because no gene information is considered, the enriched regions contain ambiguous annotations. exomePeak and MeTPeak are both exome-based peak callers that also assuming the Poisson distribution for read counts, and MeTPeak is developed based on exomePeak by integrating a hidden Markov Model (HMM). Although these two tools are exome-based, they do not process strand-specific and paired-end cases and are time consuming. Besides, with more attention on the treatment experiments of MeRIP-Seq, these tools can not satisfy the need for intuitive evaluation on the treatment effect from the comparison.

To facilitate the analysis of MeRIP-Seq, we developed “model-based analysis and inference of MeRIP-Seq (MoAIMS),” which is efficient and user-friendly software designed for transcriptome immunoprecipitation sequencing. MoAIMS can detect enriched regions and infer the signal proportion of MeRIP-Seq based on a mixture negative-binomial(NB) model. It is compatible with different RNA sequencing protocols including paired/single-end and non-strand/strand-specific sequencing. Our results demonstrated the excellent processing speed (it only takes several minutes to finish analysis of one dataset) and competitive performance of MoAIMS compared with other tools. When MoAIMS is applied to studies of m6A, the detected enriched regions contain known biological features of m6A. Furthermore, MoAIMS can provide an intuitive indicator of treatment effect for treatment experiments. The signal proportion inferred from MoAIMS for m6A treatment datasets (perturbation of m6A methyltransferases) showed a decreasing trend, consistent with experimental observations. Finally, functional analysis on the m6A perturbation datasets reveals the interplay between m6A and histone modification. In conclusion, we developed efficient and user-friendly software for MeRIP-seq analysis.

## Implementation

**Fig. 1:**
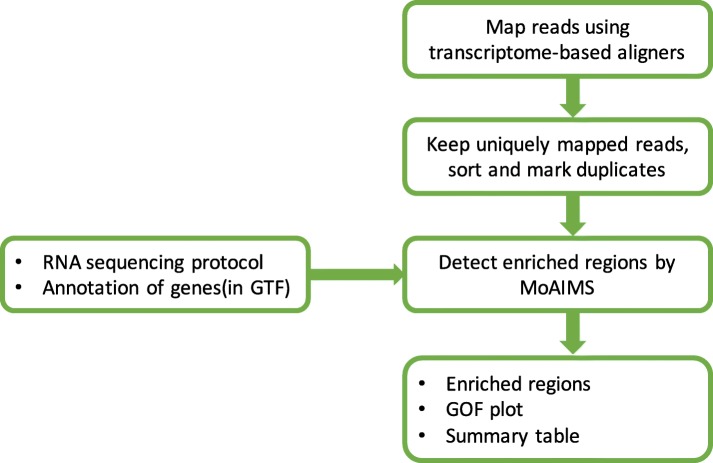
Workflow of MeRIP-Seq analysis using MoAIMS. Reads are pre-processed through alignment, sort (by coordinates), and mark-duplication. Given the RNA sequencing protocol and annotation of genes in GTF format, MoAIMS is ready for analysis. The primary outputs include detected enriched regions (in BED12 format), goodness of fitting (GOF) plots, and a model summary table

**Fig. 2:**
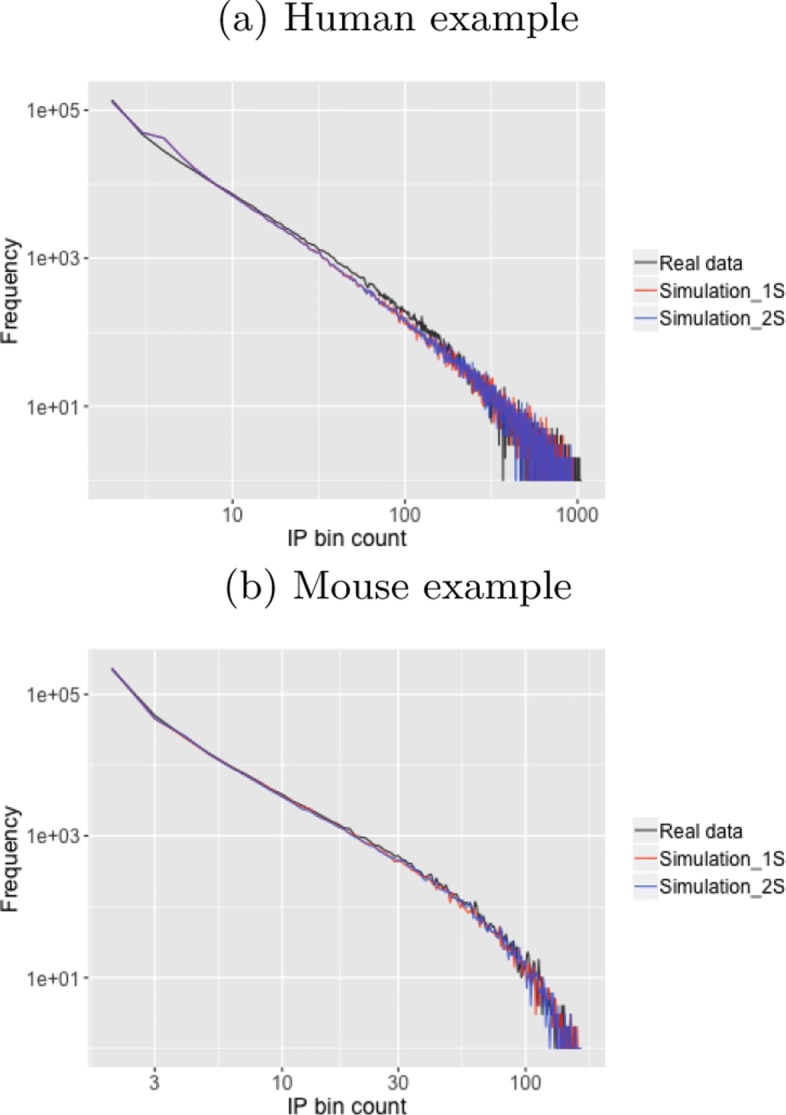
Examples of goodness of fitting (GOF) plots for a human and a mouse dataset. X-axis is bin count and Y-axis is frequency. Real data, simulation data of 1S (one-signal) mode, and simulation data of 2S (two-signal) mode, are plotted in black, red, and blue lines, respectively

A MeRIP-Seq dataset consists of one immunoprecipitation (IP) sample and one input sample (used as control). MoAIMS takes aligned IP and input bams as input. Aligned bams are generated from pre-processing as shown in the workflow of MeRIP-Seq analysis (Fig. 1). In the pre-processing, reads are aligned to a target genome by transcriptome-based aligners such as STAR [15], Tophat [16], and HISAT [17]. Only uniquely mapped reads are kept. Then, reads are sorted and marked for duplication using PicardTools [18] or samtools [19]. Given the RNA sequencing protocol (single-end or paired-end, strand-specific or not) and a target genome annotation (in GTF format), MoAIMS is ready for analysis. Typically, MoAIMS requires several minutes to complete the analysis of one MeRIP-Seq dataset. The primary outputs of MoAIMS contain enriched regions (in BED12 format), goodness of fitting (GOF) plot (Fig. 2), and a summary table of the fitted models (Table 1). The source code and the user’s manual are available at https://github.com/rreybeyb/MoAIMS

**Table 1:** An example of the model summary table

Dataset	*π*_*s*_	BIC_1S	BIC_2S	optim_k	optim_reg
WT_rep1	0.138	1679168	1678590	2	rlm
WT_rep2	0.11	1212063	1212005	2	rlm

The columns represent dataset names, signal proportion, BIC values for 1S (one-signal) mode, BIC values for 2S (two-signal) mode, optimized *k*, and optimized regression methods.

In the analysis performed by MoAIMS, it firstly obtains transcriptome bins by concatenating all exons for the expressed genes. Then, it uses featureCounts for counting reads in the bins. Subsequently, it models the distribution of the bin counts by a mixture NB distribution and detects the enriched regions. The details are described as follows.

### Read counts of bins

Counting reads in bins was performed for the transcriptome of expressed genes because unexpressed genes provide little information for signal detection. The default threshold for expressed genes is 0.5 TPM(transcripts per million). All exons for the expressed genes were concatenated and split into bins with size 200 bp(default setting). Subsequently, featureCounts [20] was used for counting reads in the bins. The parameters used in featureCounts include the following: requireBothEndsMapped=TRUE (for paired-end sequencing), read2pos=5, ignoreDup=T, allowMultiOverlap=T.

### Model construction

#### A negative-binomial mixture model

Our software implements and extends the statistical framework proposed by MOSAiCS [21], which is used to detect ChIP-Seq enriched regions and cannot be directly applied to MeRIP-Seq data because it is designed for processing DNA Sequencing and models the bin counts on the whole-genome scale. The statistical framework assumes that the observed bin counts of an IP sample follows a mixture negative-binomial model composed of a background component and a signal component that are unobserved. Let *Z* represent the components, where *Z*∈{0,1} (0 for the background component and 1 for the signal component) and *Y*_*j*_ is the observed read count of the jth bin; therefore, the mixture model can be written as Equation (1),

1 $$ {}P(Y_{j}) = {{{(1-\pi_{s})}{P(Y_{j}| Z_{j}=0,\Theta_{B})}}+{{\pi_{s}}{P(Y_{j}| Z_{j}=1,\Theta_{S})}}},  $$

of which *π*_*s*_ is the *signal proportion*(*π*_*s*_∈[0,1]), equal to *P*(*Z*_*j*_=1), and (1−*π*_*s*_) is equal to *P*(*Z*_*j*_=0); *Θ*_*B*_ and *Θ*_*S*_ are parameters of background and signal distribution respectively.

When the bin is from the background component, the read count follows the distribution $NB(a, \frac {a}{a+\mu _{j}})$, with *a* the size parameter and $\frac {a}{a+\mu _{j}}$ the probability parameter of the NB distribution. When the bin is from the signal component, the read count can be represented as *Y*_*j*_=*N*_*j*_+*S*_*j*_+*k* (one-signal, named 1S mode), where *N*_*j*_ is the count from a non-specific background following $NB(a, \frac {a}{a+\mu _{j}}), S_{j}$ is the count from an actual enrichment following $NB(b, \frac {c}{c+1})$ ($c=\frac {b}{\mu }, \mu $ is the mean), and *k* is the minimal read count required for the signal component. Thus, the distribution of the signal component is a convolution of negative binomials. Details of the distributions are provided in the Supplementary. Additionally, our software implements the mixture NB model of the signal component(two-signal, named 2S mode) from MOSAiCS considering the complexity of the signal component, where *S*_*j*_ is the count following the distribution $\pi _{s1} NB(b_{1},\frac {c_{1}}{c_{1}+1})+ (1-\pi _{s1}) NB(b_{2},\frac {c_{2}}{c_{2}+1}) $, with *π*_*s*1_ (*π*_*s*1_∈[0,1]) the first signal proportion.

In summary, the parameters of NB to be estimated in the model are represented as *Θ*={*Θ*_*B*_,*Θ*_*S*1_,*Θ*_*S*2_}, where *Θ*_*B*_=(*a*,*μ*_*j*_) for the background component, *Θ*_*S*1_=(*b,c*) for the signal component in 1S mode and *Θ*_*S*2_=(*b*_1_,*c*_1_,*b*_2_,*c*_2_) for the signal component in 2S mode.

#### Parameters estimation

First, we estimated the parameters of the background component, *Θ*_*B*_=(*a*,*μ*_*j*_). *μ*_*j*_ is estimated by regression using the input bin count data. A simple illustrative figure for the regression process is shown in Figure S1. The detailed explanation is described as follows.

Each IP bin count *Y*_*j*_ has a corresponding input bin count *X*_*j*_. For the bins from the background component, it is assumed that {*Y*_*j*_}(*j*=1,2,...,*T*) with the same input bin count from the same distribution; thus, {*Y*_*j*_} are grouped by the input bin count to $\mathcal {S}_{i}=\{Y_{j}|X_{j}=x_{i}\}$ (*x*_*i*_ is the group value equal to available and unique bin count value, i.e. 0,1,2,..., for input sample and *i* is the group index). For $Y_{j} \in \mathcal {S}_{i}$, it follows that $NB(a, \frac {a}{a+\mu _{i}})$. Subsequently, regression is performed with *x*_*i*_ as the predictor variable and *μ*_*i*_(euqal to $E(\mathcal {S}_{i})$, the median value of $Y_{j} \in \mathcal {S}_{i}$) as the response variable. MOSAiCS uses the weighted robust fitting of linear model (RLM) [22] for regression with the function log(*μ*_*i*_)=*β*_0_+*β*_1_ log(*x*_*i*_), of which *β*_0_ and *β*_1_ are the coefficients. However, in some cases of RNA sequencing, we found that the generalized additive model (GAM) [23] can provide better fitting as shown in Figure S1. GAM uses a sum of unspecified smooth functions $\sum _{s=1}^{G} f_{s}(v_{s})$ to replace the linear form $\sum _{s=1}^{G} \beta _{s} v_{s}$ in the generalized linear model where *v* is predictor variable and G is the number of predictor variables. Here, we used only one predictor variable, that is, the input bin count. Therefore, when using GAM, *μ*_*i*_ can be estimated by log(*μ*_*i*_)=*β*_0_+*f*(log(*x*_*i*_)|**β**), where *f* is represented using smoothing splines and **β** is a vector of coefficients for the spline term with length of 9 as default. We implemented GAM using R package mgcv [24] and set the restricted maximum likelihood [25] as the method for estimating the smoothing parameters. To optimize the model, MoAIMS implements both RLM and GAM and subsequently uses that with a lower BIC(Bayesian Information Criterion) [26]. BIC scores were calculated in the general method by $r\ln (T)-2\ln (\hat {L})$, where *r* is the number of parameters, *T* the number of bins, and $\hat {L}$ the maximum likelihood.

The size parameter *a* is estimated by $\hat {a}=\sum _{i} {n_{i}\hat {a_{i}}}/\sum _{i} {n_{i}}$, where $\hat {a_{i}}=[E(\mathcal {S}_{i})]^{2}/[Var(\mathcal {S}_{i})-E(\mathcal {S}_{i})]$ (the expectation is calculated using median value; the variation is calculated using the median absolute deviation) and *n*_*i*_ is the number of bins.

After estimating the parameters of the background component, expectation maximization (EM) algorithm [27] was applied to estimate the parameters of the signal component in 1S mode, *Θ*_*S*1_=(*b,c*), and *π*_*s*_. *π*_*s*_ is estimated in the maximization step with optimized *k* value rather than based on a pre-defined *k* value in MOSAiCS. For the parameters *b* and *c*, the method of moments is used as MOSAiCS. The details of modified EM process for 1S mode are provided in the Supplementary. We performed the EM process to estimate the parameters of the signal component in 2S mode, *Θ*_*S*2_=(*b*_1_,*c*_1_,*b*_2_,*c*_2_), and *π*_*s*1_ unchanged as MOSAiCS.

#### Model design for MeRIP-Seq analysis

The modification and extension of the statistical framework proposed by MoSAiCS is aimed to make our software more suitable for MeRIP-Seq analysis. This statistical framework is based on the negative-binomial distribution that is capable of modeling the variance of gene expression. We validated it by plotting the residuals between IP signal and estimated background corresponding to the gene expression. As Figure S1 shows, IP signal increases as the gene expression increases.

The modification and extension involved three aspects. First, we used log-transformation in estimating the background means instead of power-transformation in MOSAiCS because log-transformation is more commonly used in RNA sequencing analysis [28], and this can simplify the parameter tuning required in power transformation. Second, we set *k*, the minimum count in the signal regions, flexible instead of pre-defined in MOSAiCS. Because *k* may depend on the library size and signal-to-background ratio of the experiments [29], we set *k* flexible and optimized in the model fitting. With the optimized *k*, the signal proportion (*π*_*s*_) was estimated by EM rather than based on a pre-defined *k* value in MOSAiCS. Third, in addition to the RLM used by MOSAiCS in estimating background means, we applied GAM for regression to obtain better fitting for some cases of RNA sequencing data, as shown in Figure S1. An example of summary table of the fitted models is shown as Table 1 that provides signal proportion, BIC values for 1S (one-signal) mode, BIC values for 2S (two-signal) mode, optimized *k*, and optimized regression methods.

### Detection of enriched regions

The enriched regions were decided under the threshold of the false discovery rate (FDR), which was calculated as in [29, 30]. In this study, false discovery means a genomic region that is claimed to be significant when it is not. For a set $\mathcal {M}$ of *m* enriched regions that satisfies a defined cut-off (default is 0.05), the estimated FDR is equal to $(1/m) \Sigma _{j\in \mathcal {M}} P(Z=0|Y_{j}) $, where *P*(*Z*=0|*Y*_*j*_) is equal to $\frac {{(1- \hat \pi _{s})}{\hat p_{0,j}}}{{{(1- \hat \pi _{s})}{\hat p_{0,j}}}+{{ \hat \pi _{s}}{\hat p_{1,j}}}} $ for the 1S mode and $\frac {{(1- \hat \pi _{s})}{\hat p_{0,j}}}{{{(1- \hat \pi _{s})}{\hat p_{0,j}}}+{{ \hat \pi _{s}}[{\hat \pi _{s1}\hat p_{1,j}}}+(1-\hat \pi _{s1})\hat p_{1,j}]} $ for the 2S mode with $\hat p_{0,j}$ and $\hat p_{1,j}$ as the post probability for the *j*th bin from the background component and the signal component respectively. Finally, the enriched regions were merged and output in the BED12 format with the highest bin count of merged regions as the score, which can be used as a filter to obtain higher confident signal region candidates.

### Goodness of fitting (GOF)

To display the goodness of fitting (GOF), the simulations is performed using the estimated parameters. For the simulation of the 1S mode, *m* background bins and *n* signal bins were randomly sampled according to *π*_*s*_, where *m*+*n*=*T*. The background read count of *T* bins were generated from the background distribution $NB(a,\frac {a}{a+\mu _{j}}) (j=1,...,T)$. Subsequently, for *n* signal bins, the read count was composed of the background read count, the count sampled from the signal distribution $NB(b,\frac {c}{c+1})$, and the minimal count *k*. For the simulation of the 2S mode, *m* background bins, *n*1 first-signal bins, and *n*2 second-signal bins were randomly sampled according to *π*_*s*_ and *π*_*s*1_, where *m*+*n*1+*n*2=*T*. The background read count of *T* bins were generated from the background distribution $NB(a,\frac {a}{a+\mu _{j}}) (j=1,...,T)$. Subsequently, for the signal bins, the read count was composed of the background read count, the count sampled from the corresponding signal distribution $NB(b_{1},\frac {c_{1}}{c_{1}+1})$ or $NB(b_{2},\frac {c_{2}}{c_{2}+1})$, and the minimal count *k*. Figure 2 gives an example of GOF plot.

## Results

### Comparison with other tools

#### Detection of m6A-enriched regions

We performed analysis on two m6A MeRIP-Seq studies. One is from mouse embryonic stem cell [31] that uses the single-end and strand-specific sequencing protocol. The mouse datasets include the wild type and knock-out of Mettl3 (an m6A methyltransferase), of which each has two biological replicates. The other is from human A549 cell line [32] that uses the paired-end and strand-specific sequencing protocol. The human datasets contain negative control (shGFP) and perturbation of three types of m6A methyltransferases including Mettl14, Mettl3, and WTAP, of which each has two replicates. Table S1 summarized the information of datasets. Raw fastq files were retrieved from Gene Expression Omnibus [33] with accession numbers GSE52662 and GSE54365. Reads were aligned to human (hg19) and mouse (mm10) genome using STAR (version 2.6.0c, default setting) [15] with annotation files of GENCODE (human release19 and mouse release M19) [34]. Only uniquely mapped reads were kept. The sorted (by coordinates) and duplication-marked bam files were generated by Picard (version 2.18.1) and subsequently used as input for MoAIMS.

Three commonly-used tools for comparison are MACS(version MACS2), exomePeak(v2.13.2) and MeTPeak(v1.0.0). Duplication-removed bam files were used as input for the three tools. For MACS, we specified parameters “–nomodel –extsize=100 –keep-dup=all -g 286,000,000 (for human)/221,000,000 (for mouse)’. ’We kept the peaks called by MACS overlapped with exonic regions for comparison. For exomePeak and MeTPeak, we used the default setting.

First, we compared the m6A-enriched regions called by MoAIMS with MACS, exomePeak, and MeTPeak. We verified to what extent the enriched regions called by the four tools agree with each other using BEDTools [35]. To obtain higher confident regions, we chose the enriched regions (FDR ≤0.05) called by MoAIMS with score ≥10. Table S2 shows the results for the mouse wild-type datasets. Each cell of the table represents the percentage of enriched regions of tools in the columns detected by tools in the rows; the number in bracket is the number of enriched regions called by each tool. It is indicated that our enriched regions are overlapped more with MACS and exomePeak. Additionally, MeTPeak called relatively less peaks and, in some cases, could miss enriched regions, as shown in Figure S1.

**Fig. 3:**
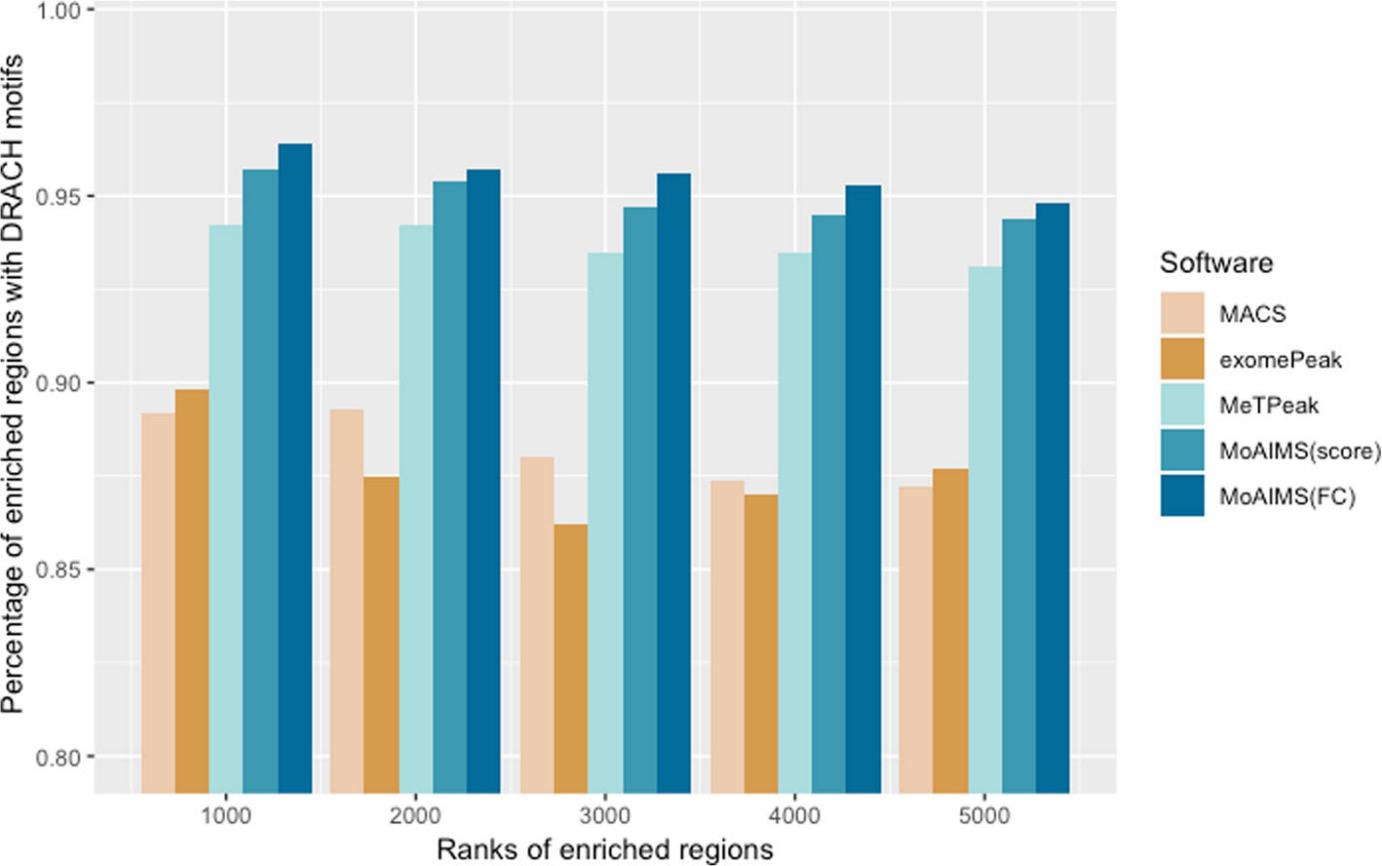
Comparison of motif occurrence for MACS, exomePeak, MeTPeak, and MoAIMS for a wild-type mouse dataset. The X-axis is the decreasing rank of the enriched regions from the top 1000 to top 5000. The ranking scheme for MACS, exomePeak and MeTPeak is fold change. For MoAIMS, the ranking scheme of fold change(FC) and score are both used for comparison. The Y-axis is the percentage of motif occurrence

Subsequently, we verified the occurrence of the DRACH motif [36], a classic m6A motif where D = A, G, or U; R = A or G; and H = A, C, or U, in the top-5000 enriched regions. The ranking scheme for MACS, exomePeak and MeTPeak is fold change. For MoAIMS, the ranking scheme of fold change(FC) and score are both used for comparison. Sequences of length 200 bp were extracted around the summits of the enriched regions. For MACS, we used the summits it provided; for MoAIMS, exomePeak, and MeTPeak, the summits were defined as the positions with the highest read coverage. Because we had the strand-specific sequencing data, we only counted the motifs that occurred in the expressed genes with coverages (for MACS, only motifs with coverages were counted). Figure 3 compares the percentage of motif occurrence in the decreasing peak ranks for a wild-type mouse dataset (comparisons are also conducted for the other untreated datasets shown in Figure S1). The results indicated that our software achieved comparable performance to the other three tools.

**Fig. 4:**
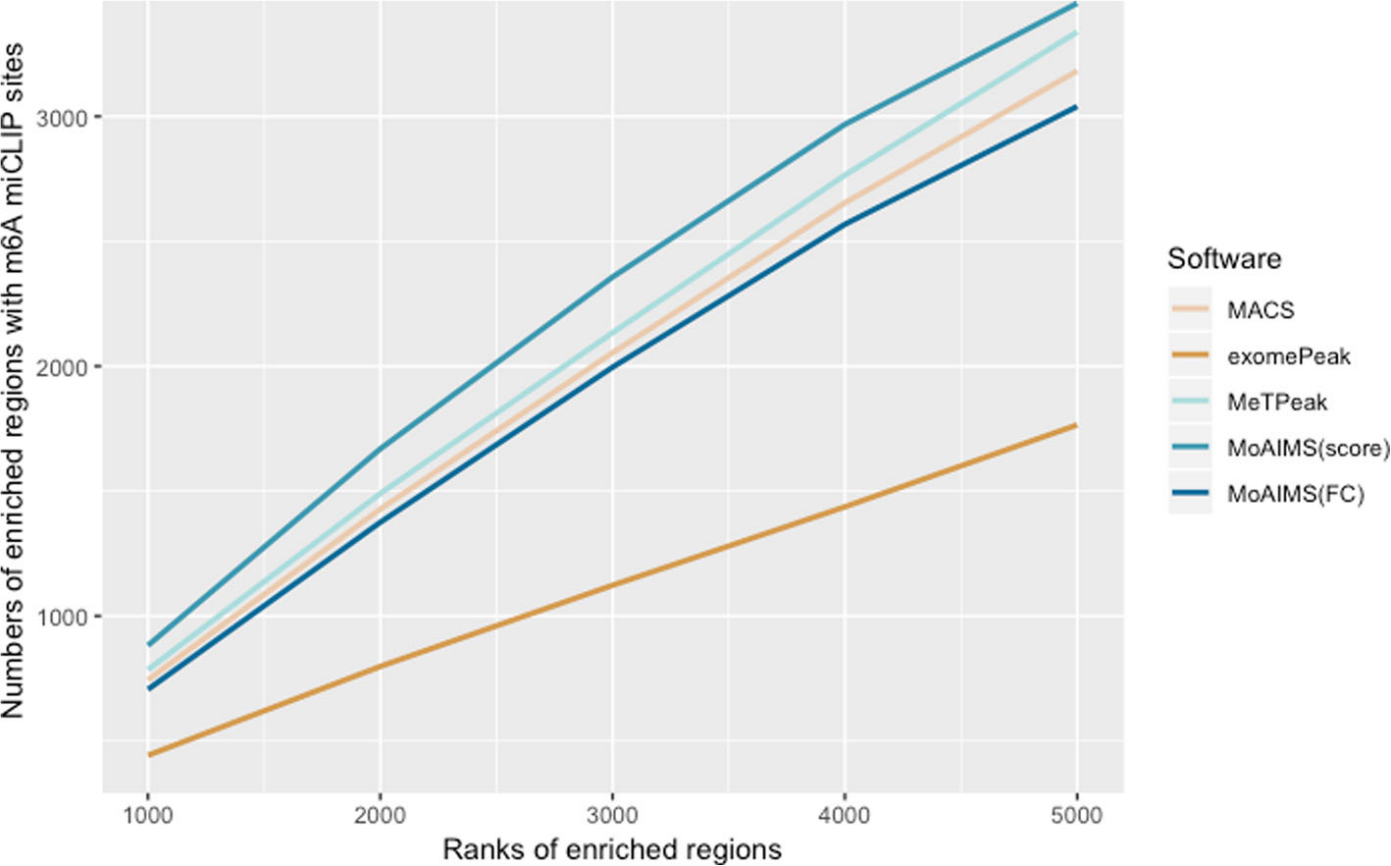
Comparison of top enriched regions with m6A miCLIP sites called by MACS, exomePeak, MeTPeak, and MoAIMS for a human negative control dataset. X-axis is the decreasing ranks of the enriched regions from the top 1000 to top 5000. The ranking scheme for MACS, exomePeak and MeTPeak is fold change. For MoAIMS, the ranking scheme of fold change(FC) and score are both used for comparison. Y-axis is the number of enriched regions with m6A miCLIP sites

Next, we are interested to know to what extent the m6A miCLIP sites agree with the MeRIP-Seq enriched regions. We collected miCLIP-Seq data of human A549 cell line from [37], which maps m6A sites at single-base resolution.We counted the number of regions containing miCLIP sites in the top-5000 enriched regions detected by the four tools(The ranking scheme is the same as that for counting motif occurrence). Figure 4 shows that our software with score ranking has the most number of regions with m6A miCLIP sites in the decreasing peak ranks (comparisons were also conducted for the other human dataset provided in Figure S1). To determine whether the number was affected by the length of the enriched regions, we compared the length of the top-5000 enriched regions between the tools, as shown in Table S3. The result shows that compared with MeTPeak, which ranks second with regard to consistency with miLCIP sites, MoAIMS can detect more regions with m6A miCLIP sites under the similar resolution.

#### Features of MoAIMS

MoAIMS is efficient software with appealing features, as shown in Table 2. Thus, we performed comparison analysis with regard to those features. First, because our software is compatible with general RNA sequencing protocols in counting reads, we investigated how the methods of counting reads affected the detection of enriched regions for pair-end RNA sequencing. The comparison was conducted for the human shGFP (negative control) datasets among exome-based callers: MoAIMS, exomePeak, and MeTPeak. Table S4 lists the number of enriched regions detected by these three tools using pair-end reads and first-in-pair reads, separately. The result indicates that exomePeak and MeTPeak differ in the method of counting paired-end reads, while the difference is limited for our software.

**Table 2:** Features of MoAIMS compared with other tools

Features	MoAIMS	exomePeak	MeTPeak	MACS
Exome-based	Y	Y	Y	N
Strand-specific/Paired-end	Y	N	N	N
Time-consuming	N	Y	Y	N
Inference of signal proportion	Y	N	N	N
Visualization of model fitting	Y	N	N	N
Output in BED12 format	Y	Y	Y	N
Support for differential methylation analysis	N	Y	N	N

**Fig. 5:**
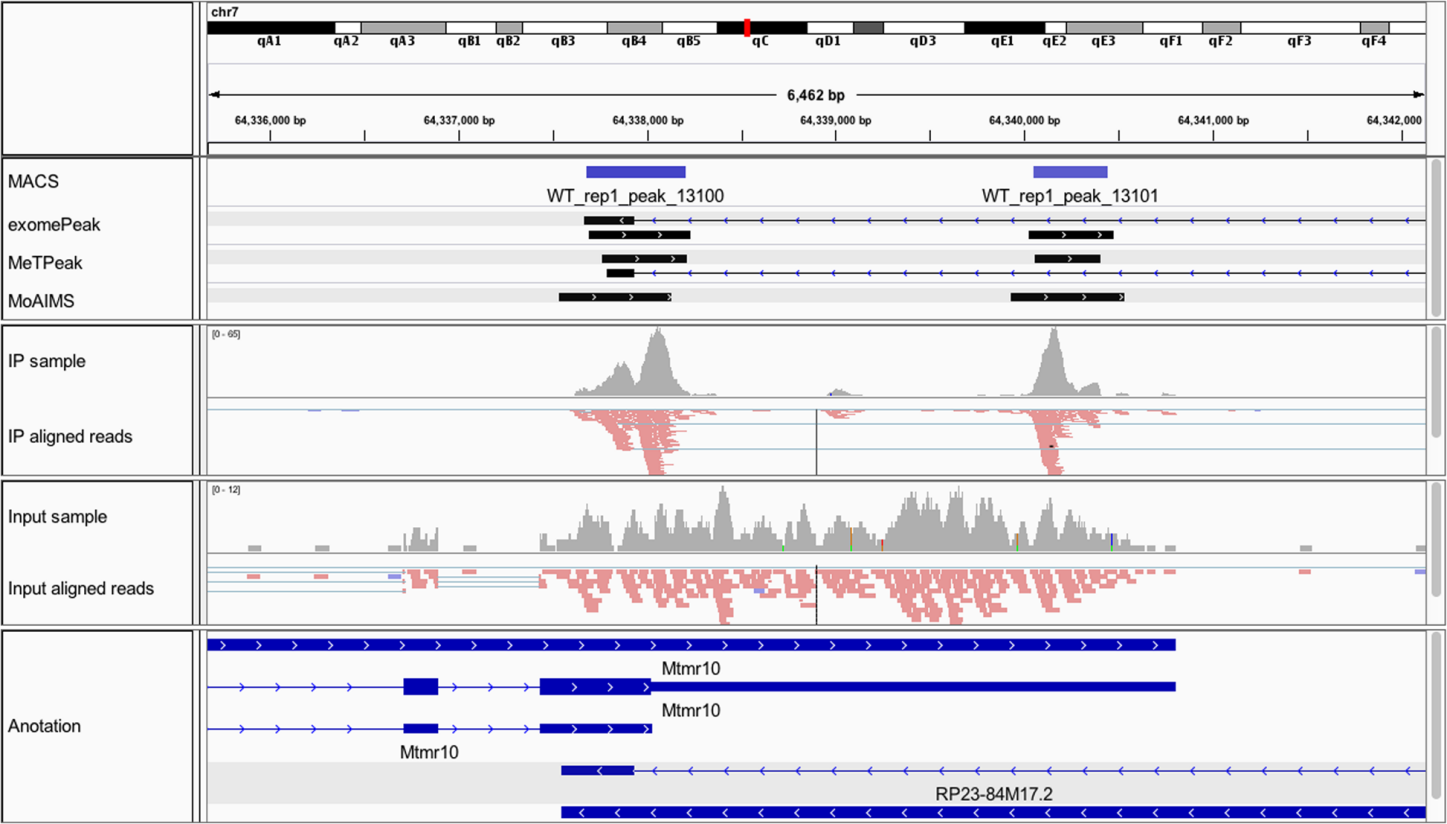
Example of detection of strand-specific enriched regions. The plot is generated using IGV [38], showing the enriched region called by MACS, exomePeak, MeTPeak, and MoAIMS in the first four tracks. The following tracks are coverage and aligned reads (strand orientation is colored) for the IP and input sample, respectively, and the genome annotation

Next, our software is a strand-aware caller; thus, it can avoid calling ambiguous regions that are overlapped with other regions on different strands. Figure 5 shows an example of how our software called strand-specific enriched regions. As shown in the figure, a protein-coding gene Mtmr10 and an antisense gene RP23-84M17.2 are partially overlapped. The coverage track in red (colored by strand) indicates the signal in Mtmr10, not the antisense gene. For this case, exomePeak and MeTPeak have callings on both genes, but MoAIMS can avoid the ambiguous callings.

Finally, our software offers excellent processing speed compared with exome-based callers exomePeak and MeTPeak, which require approximately 2 hours to analyze one dataset (MeTPeak needs even more time because it applies HMM). Table 3 lists the time cost for a human and a mouse dataset, indicating that our software is competitive as it only requires several minutes and can yield comparable performance.

**Table 3:** Performance on the time cost

Dataset	MoAIMS	exomePeak	MeTPeak
Human shGFP_rep1	14.1	141.0	176.4
Mouse WT_rep1	10.6	110.4	143.4

shGFP_rep1 is one human negative control dataset. WT_rep1 is one wild-type mouse dataset. The units of time is minute.

### Application on feature and functional analysis of m6A

**Fig. 6:**
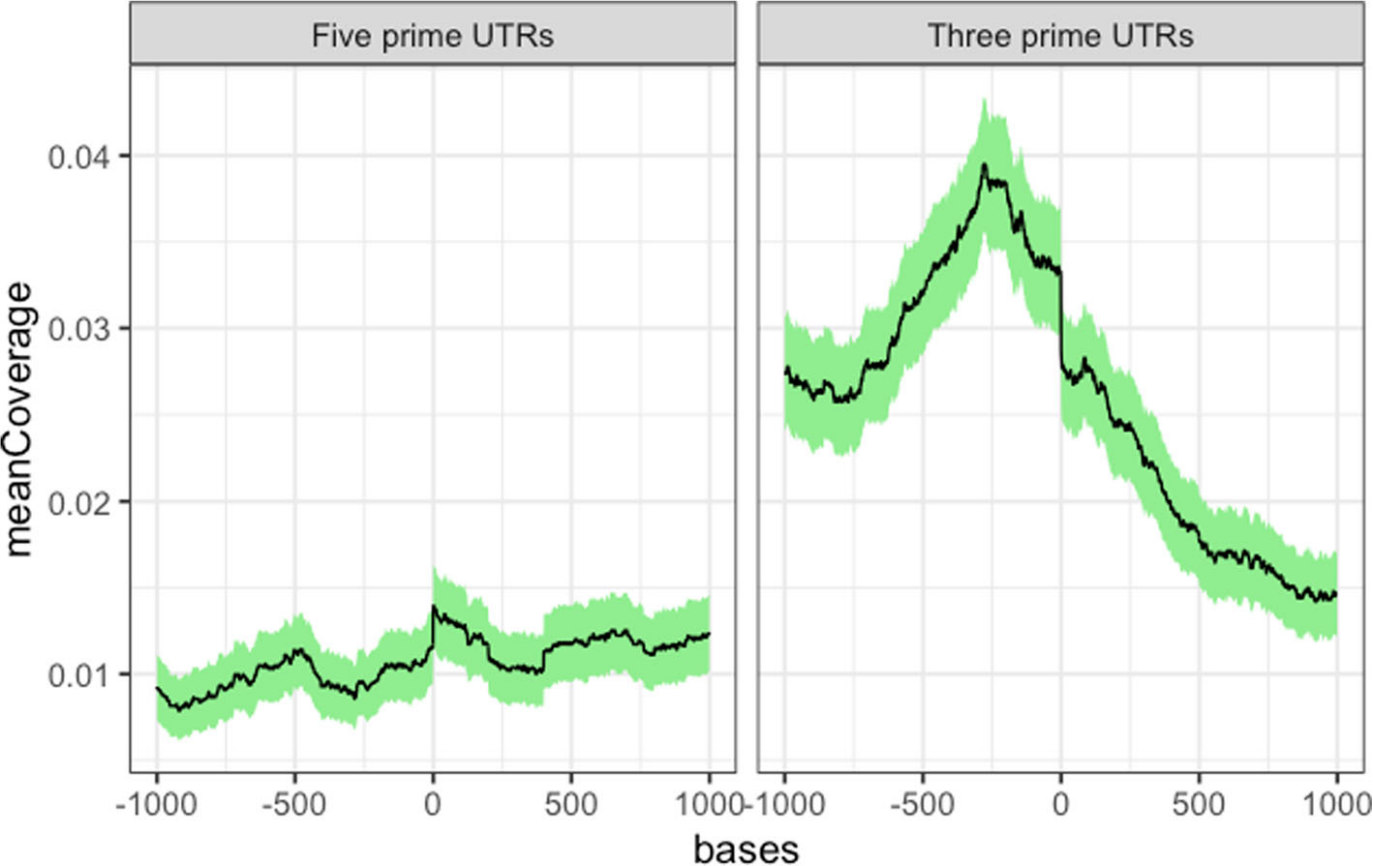
Position profile of m6A-enriched regions for a wild-type mouse dataset. X-axis is the relative position coordinates and Y-axis is the mean coverage of the enriched regions. The plot is generated using RCAS [39]

m6A is characterized by its location preference close to three prime untranslated regions (3’ UTRs); thus, we verified the position preference of the enriched regions (with score ≥10) called by MoAIMS. For the wild-type mouse datasets, as shown in Figure 6 and S7(a), the enriched regions exhibit location bias near 3’ UTRs, which is consistent with the results of the original study [31]. For the human negative control datasets, we observed that enriched regions appeared near 5’ UTRs, as shown in Figures S7(b) and (c), which agrees with the findings of the original study [32] regarding methylated m6A at transcription start sites.

**Fig. 7:**
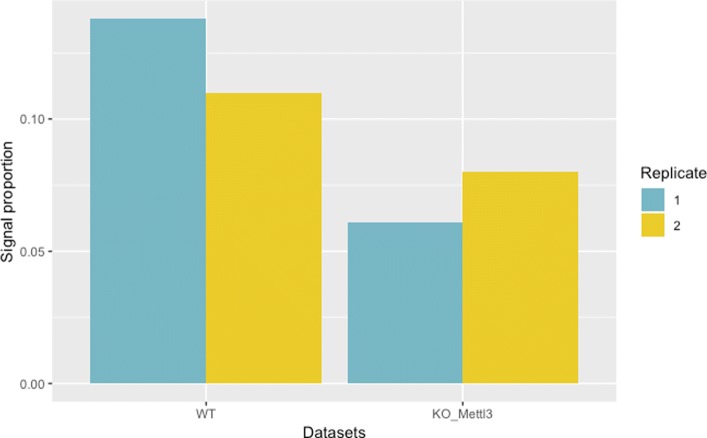
Signal proportion for m6A treatment experiments. X-axis represents two mouse MeRIP datasets of wild type (WT) and knock-out of METT13 (KO_Mettl3) with blue for replicate 1 and yellow for replicate 2. Y-axis represents the signal proportion

Because our software infers the signal proportion from the mixture NB model, we assumed that this value can reflect the treatment effect; for example, the knocking-down/out of methyltransferases (such as WTAP, METTL3, or METTL14) can cause decreased signal proportion. For the mouse datasets, as shown in Fig. 7, Mettl3 knock-out exhibits a clear decreasing trend for signal proportion, which agrees with the findings of a recent study [40] that include a discussion on the m6A methyltransferase treatment experiments and the effect of treatment in this dataset. For the human datasets, as shown in Figure S1, WTAP shows a relatively clear effect after perturbation, while Mettl3 and Mettl14 shows less effect. This trend is consistent with the original study [32], in which the authors observed the necessity of WTAP for m6A methylation, while perturbation of Mettl3 and Mettl14 exhibited milder effects in decreasing methylation level. These results suggest that the signal proportion can be used as an intuitive indicator of the m6A treatment effect, which can facilitate biologists’ evaluation on the treatment experiments.

**Fig. 8:**
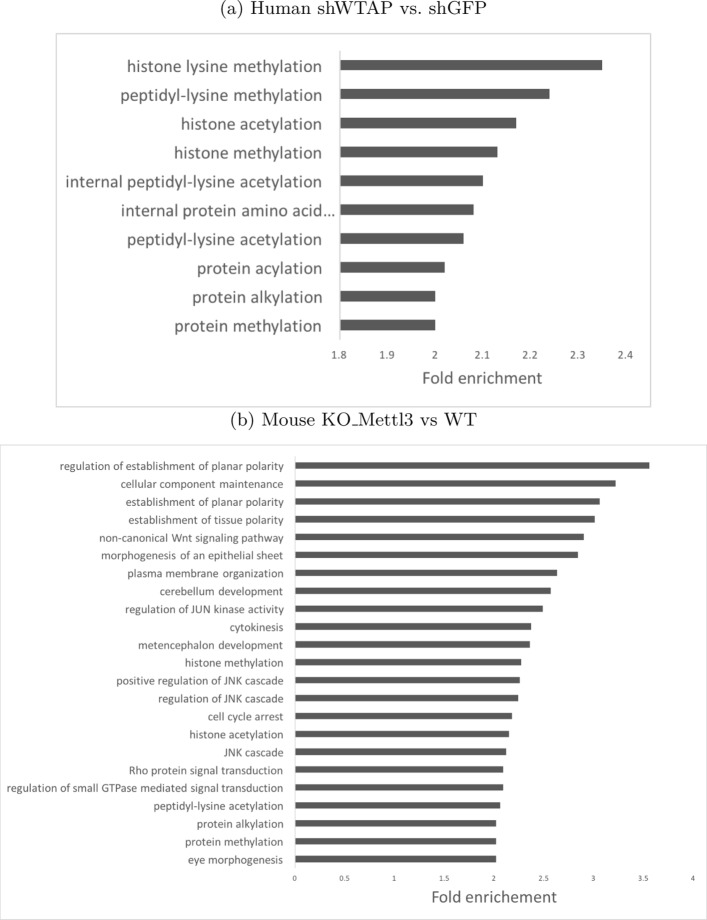
Enriched biological process (BP) term for genes impacted by perturbation of m6A methyltransferase for shWTAP vs. shGFP(Human) and KO_Mettl3 vs WT(Mouse). The threshold of the adjusted p-value for the terms are set as 0.05

Finally, we conducted a functional analysis on the genes affected by the perturbation of methyltransferases. We performed gene ontology (GO) analysis by RCAS [39] on genes with lost m6A-enriched regions. The loss of m6A-enriched regions is defined as a state from being detected in all the replicates of the wild type to being undetected in all the replicates of the treated type. The GO results of enriched biological process (BP) terms are shown in Fig. 8. For the mouse datasets of the wild type and Mettl3 knock-out, the enriched BP terms are related to planar polarity and polarity, thus suggesting that the loss of m6A affects the development of embryo cells. For the human datasets of negative control and WTAP perturbation, the enriched BP terms are related to histone methylation and acetylation, which also appeared in the term list for mouse. This observation agrees with that of [41] regarding m6A’s function in destabilizing transcripts that encode histone modification enzymes.

## Discussion

MoAIMS is an efficient and user-friendly software for the analysis of MeRIP-Seq. Nonetheless, improvements are still required. First, MoAIMS currently supports only the analysis of single samples. For replicate samples, although enriched regions common in all the replicates can be easily extracted using our software, a joint statistical model can be developed as an alternative that considers the variance among replicates. Next, apart from the NB distribution, other statistical distributions are worth being tested owing to the wide diversity of RNA sequencing data. For example, Poisson–Tweedie has been proposed for studying differential expressed genes as it is a more general family of count data distributions that can fit RNA sequencing data under situations of heavy tail or zero inflation [42]. Additionally, the double Poisson distribution has been applied to manage under-dispersion RNA sequencing data [43]. Last but not least, because our software can provide user-friendly outputs for downstream analysis, it is feasible to integrate MeRIP-Seq datasets with other biological data for a comprehensive functional analysis, especially for MeRIP-Seq-treatment experiments.

## Conclusion

We developed MoAIMS, which is an efficient and user-friendly software for analysis of MeRIP-Seq. MoAIMS is compatible with general RNA sequencing protocols, achieves excellent speed and competitive performance, and provides user-friendly outputs for downstream analysis. When MoAIMS was applied to studies of m6A, m6A’s known biological features and its interplay with histone modification was revealed. Furthermore, the signal proportion inferred from MoAIMS can be used as an intuitive indicator of treatment effect. We hope that MoAIMS would facilitate MeRIP-Seq analysis and provide more insights into studies of RNA modification.

## Availability and requirements

- **Project name:** MoAIMS
- **Project home page:** https://github.com/rreybeyb/MoAIMS
- **Operating systems:** Linux, Mac OS, Windows
- **Programming language:** R
- **Other requirements:** R version 3.4.0 or higher
- **License:** GNU GPL
- **Any restrictions to use by non-academics:** None

## Supplementary information

**Additional file 1** Supplementary materials for "MoAIMS: efficient software for detection of enriched regions of MeRIP-Seq".

## Data Availability

The datasets and materials can be downloaded from https://github.com/rreybeyb/MoAIMS.

## Abbreviations

3’ UTRs: three prime untranslated regions
BIC: bayesian information criterion
BP: biological process
ChIP-Seq: Chromatin immunoprecipitation sequencing
EM: expectation maximization
FDR: false discovery rate
GAM: generalized additive model
GO: gene ontology
GOF: goodness of fitting
HMM: hidden markov model
IP: immunoprecipitation
m1A: N1-methyladenosine
m5C: 5-methylcytidine
m6A: N6-methyladenosine
MeRIP-Seq: Methylated RNA immunoprecipitation sequencing
NB: negative-binomial
NGS: next-generation sequencing
RLM: robust fitting of linear models
TPM: transcripts per million
