# Supplementary material for: MoAIMS: efficient software for detection of enriched regions of MeRIP-Seq
Source: BMC Bioinformatics. 2020 Mar 14;21:103. doi: 10.1186/s12859-020-3430-0 (PMC7071693; doi:10.1186/s12859-020-3430-0)
Supplement: Supplementary file 1 — Additional file 1 Supplementary materials for "MoAIMS: efficient software for detection of enriched regions of MeRIP-Seq". [file 12859_2020_3430_MOESM1_ESM.pdf]

Supplementary materials for “MoAIMS: Efficient Software for Detection of Enriched Regions of MeRIP-Seq”

Supplementary text

Negative-Binomial distribution

A negative binomial distribution is defined as $NB(r, p)$ with two parameters r and p, representing size and probability, respectively. The density function is

$$P(Y = y) = \frac{\Gamma(y + r)}{y!\Gamma(r)} p^r (1 - p)^y \tag{S1.1}$$

with $y = 0, 1, 2, ..., r > 0$ and $0 < p \leq 1$.

As p can be represented by $p = \frac{r}{r+\mu}$, where μ is the mean, the density function can be written as

$$P(Y = y) = \frac{\Gamma(y + r)}{y!\Gamma(r)} \left(\frac{r}{r + \mu}\right)^r \left(\frac{\mu}{r + \mu}\right)^y . \tag{S1.2}$$

Implementation and extension of 1S mode of MoSAICS[1]

Our software implements and extends the statistical framework proposed by MOSAiCS. The followings provide details of modified 1S mode of MOSAiCS.

A MeRIP-Seq dataset consists of one (IP) sample and one input sample. It is assumed that the observed bin counts of an IP sample follow a mixture (NB) model composed of a background component and a signal component that are unobserved. Let Z represent the components, where $Z \in \{0, 1\}$(0 for the background component and 1 for the signal component), and Y_j is the observed read count of the jth bin; therefore, the mixture model can be written as the following equation,

$$P(Y_j) = (1 - \pi_s)P(Y_j|Z_j = 0, \Theta_B) + \pi_s P(Y_j|Z_j = 1, \Theta_S), \tag{S2}$$

where π_s is the *signal proportion*($\pi_s \in [0, 1]$), equal to $P(Z_j = 1)$, and $(1 - \pi_s)$ is equal to $P(Z_j = 0)$; Θ_B and Θ_S are parameters of background and signal distribution respectively.

When the bin is from the background component, the read count follows the distribution $NB(a, \frac{a}{a+\mu_j})$ which can be written as

$$P(Y_j = y|Z_j = 0) = \frac{\Gamma(y + a)}{y!\Gamma(a)} \left(\frac{a}{a + \mu_j}\right)^a \left(\frac{\mu_j}{a + \mu_j}\right)^y . \tag{S3}$$

When the bin is from the signal component, the read count can be represented as $Y_j = N_j + S_j + k$, where N_j is the count from a non-specific background following $NB(a, \frac{a}{a+\mu_j})$ as defined in Equation(S3), S_j is the count from an actual enrichment following $NB(b, \frac{c}{c+1})(c = \frac{b}{\mu}$,$\mu$ is the mean), and k is the minimal read count required for the signal component. Thus, the distribution of the signal component is a convolution of negative binomials. The convolution of two discrete distributions is defined as $P(X = X_1 + X_2) = P(X_1) * P(X_2) = \sum_{n=0}^{x} P_1(n)P_2(x-n)$, when X_1 and

X_2 are two random variables with distributions $P_1(X_1)$ and $P_2(X_2)$, respectively; therefore, the distribution of the signal component can be written as

$$\begin{aligned} &P(Y_j = y-k|Z_j=1) \\ &\quad = P(S_j) * P(N_j) \\ &\quad = \sum_{q=0}^{y-k}\left[\frac{\Gamma(y-k-q+b)}{(y-k-q)!\Gamma(b)}\left(\frac{c}{c+1}\right)^b\left(\frac{1}{c+1}\right)^{(y-k-q)}\right]\left[\frac{\Gamma(q+a)}{q!\Gamma(a)}\left(\frac{a}{a+\mu_j}\right)^a\left(\frac{\mu_j}{a+\mu_j}\right)^q\right]. \end{aligned} \tag{S4}$$

For estimating the parameters, a and μ_j of the background component are estimated by regression using the input bin counts, while b and c of the signal component and π_s are estimated by expectation maximization(EM).

Each IP bin count Y_j has a corresponding input bin count X_j. For the bins from the background component, it is assumed that $\{Y_j\}(j=1,2,...,T)$ with the same input bin count from the same distribution; thus, $\{Y_j\}$ are grouped by the input bin count to $\mathcal{S}_i = \{Y_j|X_j = x_i\}$($x_i$ is the group value equal to available and unique input bin count value(0,1,2,...) and i is the group index). For $Y_j \in \mathcal{S}_i$, it follows that $NB(a, \frac{a}{a+\mu_i})$. Let μ_i be $E(\mathcal{S}_i)$ (the median value of $Y_j \in \mathcal{S}_i$); then, the regression is fitted through RLM or GAM as Equations (S5) and (S6),

$$\log(\mu_i) = \beta_0 + \beta_1 \log(x_i), \tag{S5}$$

where β_0 and β_1 are coefficients, and

$$\log(\mu_i) = \beta_0 + f(\log(x_i)|\boldsymbol{\beta}), \tag{S6}$$

where f is represented using thin plate regression splines and $\boldsymbol{\beta}$ is a vector of coefficients for the spline term with length of 9 as default. The regression method is optimized in MoAIMS based on the BIC value.

a is estimated by $\hat{a} = \sum_i n_i\hat{a}_i / \sum_i n_i$, where $\hat{a}_i = [E(\mathcal{S}_i)]^2/[Var(\mathcal{S}_i) - E(\mathcal{S}_i)]$ (the expectation is calculated using the median value; the variation is calculated using the median absolute deviation) and n_i is the number of bins.

The estimations of π_s, b, and c using EM are shown as follows with the initiation values for π_s, b, and c set empirically to 0.02, 0.2, and 2, respectively. Because this algorithm employed various approximated estimations for efficient calculation, EM can not ensure the monotonic increase of likelihood. We set the initiation value of the signal proportion low enough so that it is expected to be closer to the real value after each iteration.

The complete data likelihood can be written as Equations (S7),(S8), where T is the number of bins and $I(Z)$ is the indicator function,

$$L = \Pi_{j=1}^{T}[(1-\pi_s)P(Y_j|Z_j=0,\Theta_B)]^{I(Z_j=0)} + [\pi_s P(Y_j|Z_j=1,\Theta_S)]^{I(Z_j=1)}, \tag{S7}$$

$$\log L = \sum_{j=1}^{T} [I(Z_j = 0)(\log(1 - \pi_s) + \log P(Y_j|Z_j = 0, \Theta_B)) \\ + I(Z_j = 1)(\log \pi_s) + \log P(Y_j|Z_j = 1, \Theta_S))]. \tag{S8}$$

The expected complete data likelihood is

$$Q = \sum_{j=1}^{T} [P(Z_j = 0|Y_j)(\log(1 - \pi_s) + \log P(Y_j|Z_j = 0, \Theta_B)) \\ + P(Z_j = 1|Y_j)(\log \pi_s + \log P(Y_j|Z_j = 1, \Theta_S))]. \tag{S9}$$

E-step:

$$z_{1,j}^{(t)} = P(Z_j = 1|Y_j) \\ = \frac{\pi_s^{(t)} P(Y_j|Z_j = 1, \Theta_S^{(t)})}{(1 - \pi_s^{(t)}) P(Y_j|Z_j = 0, \Theta_B^{(t)}) + \pi_s^{(t)} P(Y_j|Z_j = 1, \Theta_S^{(t)})}, \tag{S10}$$

$$z_{0,j}^{(t)} = P(Z_j = 0|Y_j) \\ = 1 - z_{1,j}^{(t)}. \tag{S11}$$

M-step:

For the parameter π_s, to maximize the expected log likelihood with respect to π_s, we obtained

$$\frac{\partial Q}{\partial \pi_s} = \frac{\sum_{j=1}^{T} P(Z_j = 0|Y_j)}{1 - \pi_s} - \frac{\sum_{j=1}^{T} P(Z_j = 1|Y_j)}{\pi_s} = 0. \tag{S12}$$

Solving Equation (S12), we obtained

$$\pi_s^{(t+1)} = \frac{1}{T} \sum_{j=1}^{T} z_{1,j}^{(t)}. \tag{S13}$$

For the parameters b and c, the method of moments is used by utilizing

$$Var(S_j) = Var(Y_j|Z_j = 1) - Var(N_j) \\ = E(S_j) + \frac{E(S_j)^2}{b^{(t)}}, \tag{S14}$$

$$c^{(t)} = \frac{b^{(t)}}{E(S_j)}. \tag{S15}$$

Solving Equations (S14) and (S15), we obtained

$$b^{(t+1)} = \frac{E(S_j)^2}{Var(Y_j|Z_j = 1) - Var(N_j) - E(S_j)}, \tag{S16}$$

$$c^{(t+1)} = \frac{E(S_j)}{Var(Y_j|Z_j = 1) - Var(N_j) - E(S_j)}, \tag{S17}$$

where $E(S_j) = E(Y_j|Z_j = 1) - E(N_j) - k$. We calculate $E(Y_j|Z_j = 1), Var(Y_j|Z_j = 1), E(N_j)$ and $Var(N_j)$ by,

$$\begin{aligned}
E(Y_j|Z_j = 1) &= \frac{\sum_{j=1}^{T} z_{1,j}^{(t)} Y_j}{\sum_{j=1}^{T} z_{1,j}^{(t)}}, \\
Var(Y_j|Z_j = 1) &= \frac{\sum_{j=1}^{T} z_{1,j}^{(t)} [Y_j - E(Y_j|Z_j = 1)]^2}{\sum_{j=1}^{T} z_{1,j}^{(t)}}, \\
E(N_j) = \hat{\mu_0} &= \frac{\sum_{j=1}^{T} \hat{\mu}_j}{T}, \\
Var(N_j) &= \hat{\mu_0}(1 + \hat{\mu_0}/\hat{a}),
\end{aligned}$$

in that $E(Y_j|Z_j = 1)$ and $Var(Y_j|Z_j = 1)$ are the weighted mean and variance, respectively; $E(N_j)$ and $Var(N_j)$ are calculated using the method of moments with $\hat{\mu}_j$ and $\hat{a}$ estimated from the previous steps, of which $\hat{\mu}_j$ is equal to $\frac{\sum_{j=1}^{T} \exp[\hat{\beta}_0 + \hat{\beta}_1 \log(x_j)]}{T}$ using RLM or $\frac{\sum_{j=1}^{T} \exp[\hat{\beta}_0 + f(\log(x_j)|\hat{\boldsymbol{\beta}})]}{T}$ using GAM.

Supplementary figures and tables

Gene1:IP	1	2	5	1	1
Gene1:input	3	3	3	3	3

Gene2:IP	2	2	3	2
Gene2:input	6	6	6	6

Steps

- Group IP count(Y) according to input count(X), x_i is the group value:
  $$\mathcal{S}_1 = \{Y = 1, 2, 5, 1, 1 | X = x_1 = 3\}$$
  $$\mathcal{S}_2 = \{Y = 2, 2, 3, 2 | X = x_2 = 6\}$$
- Get median Y value of each group
  $$\mu_1 = E(\mathcal{S}_1) = 1$$
  $$\mu_2 = E(\mathcal{S}_2) = 2$$
- To estimate the mean background count of each bin from IP sample, regression is performed using x_i as the predictor variable and $\mu_i$as the response variable

Figure S1 A simple illustration on estimating background means. Tables list the IP and input bin counts for Gene1 and Gene2 as examples. Cells with numbers represent bins. The followeing steps show how to estimate the mean background count of each bin from IP sample in brief.

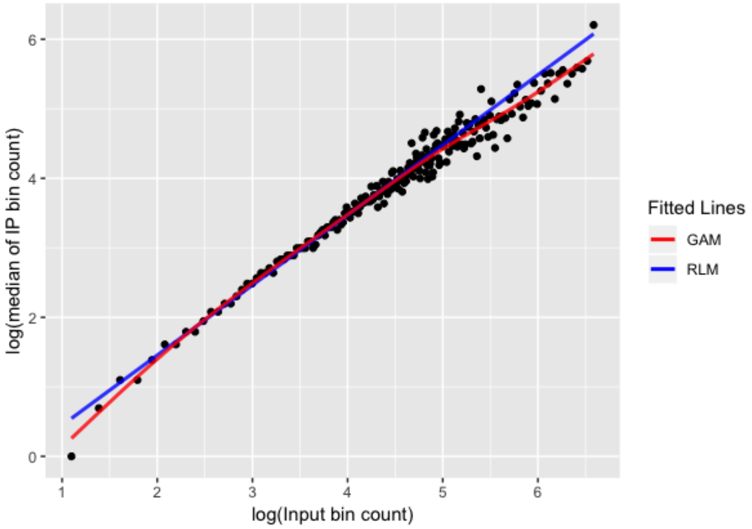

Figure S2 Comparison of generalized additive model (GAM) and robust fitting of linear model (RLM) regression for estimating the distribution means of the background component. X-axis is the available and unique read count for input sample and Y-axis is the median read count of the IP bins of which corresponding input bins have the same read count. Both are log transformed.

(a) Human shGFP_rep1

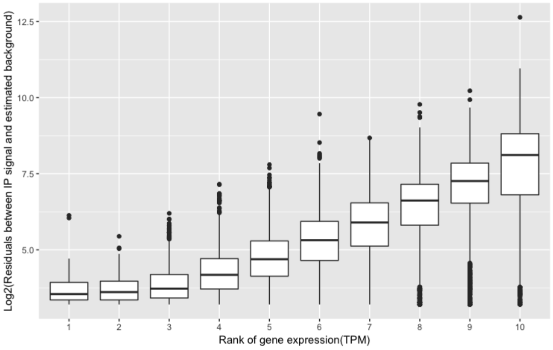

(b) Mouse WT_rep1

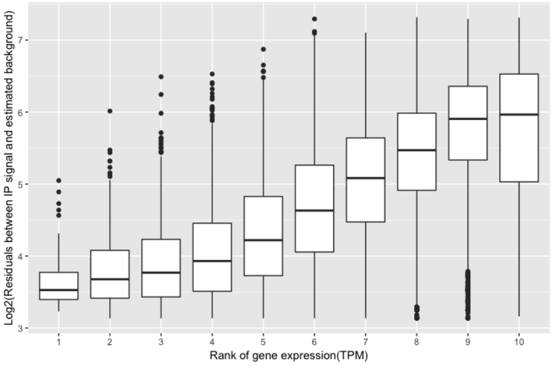

Figure S3 Diagnostic plots of a human dataset shGFP_rep1 and a mouse dataset WT_rep1 for the residuals between IP signal and estimated background corresponding to the gene expression. X-axis is the increasing rank of gene expression(TPM). Y-axis is the residuals between IP signal and estimated background in log2 scale.

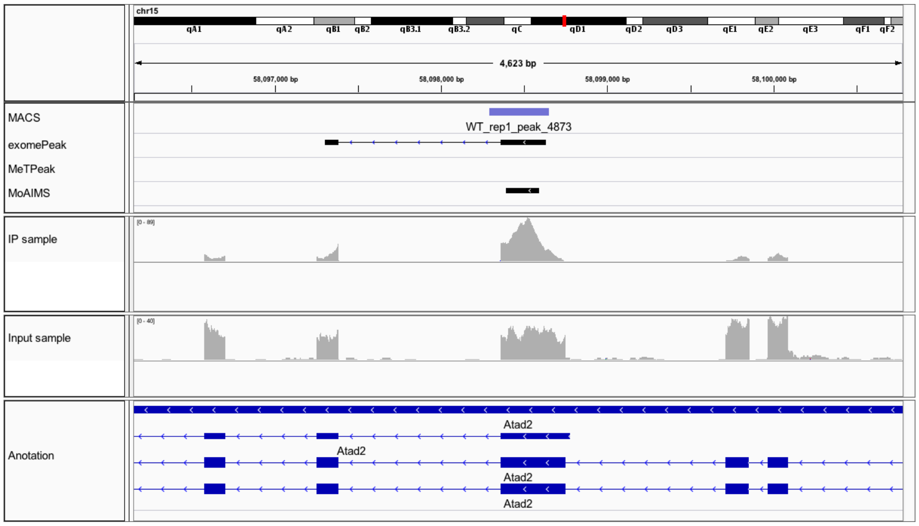

Figure S4 Example of an enriched region missed by MeTPeak. The plot is generated using IGV showing the enriched region called by MACS, exomePeak, MeTPeak, and MoAIMS in the first four tracks. The following tracks are the coverage for IP and input sample and the genome annotation.

(a) Mouse WT_rep2

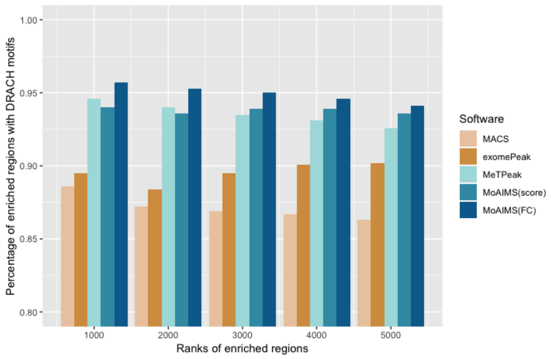

(b) Human shGFP_rep1

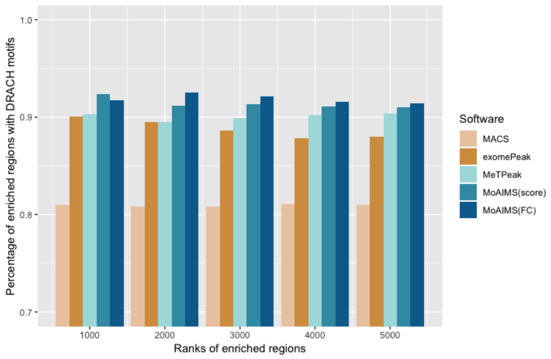

(c) Human shGFP_rep2

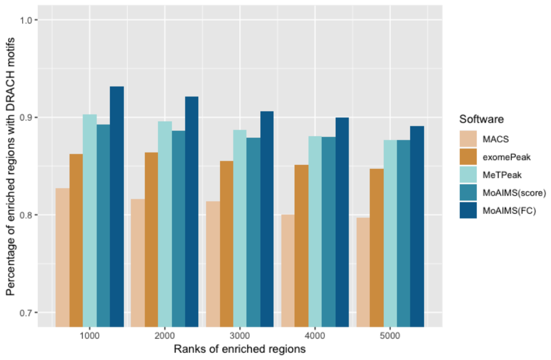

Figure S5 Comparison of motif occurrence for MACS, exomePeak, MeTPeak, and MoAIMS for datasets: WT_rep2(wild-type mouse replicate 2), shGFP_rep1 and shGFP_rep2 (two replicates of human negative control). X-axis is the decreasing rank of the enriched regions from the top 1000 to top 5000. The ranking scheme for MACS, exomePeak and MeTPeak is fold change. For MoAIMS, the ranking scheme of fold change(FC) and score are both used for comparison. Y-axis is the percentage of motif occurrence.

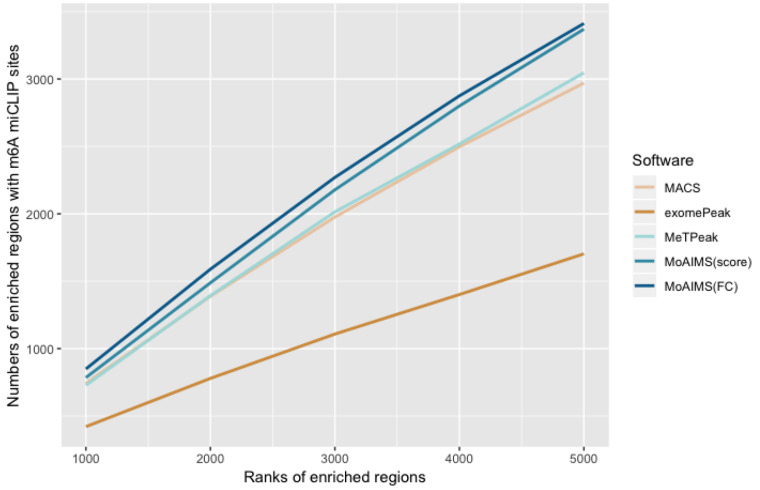

Figure S6 Comparison of top enriched regions with m6A miCLIP sites called by MACS, exomePeak, MeTPeak, and MoAIMS for the other human negative control dataset. X-axis is the decreasing rank of the enriched regions from the top 1000 to top 5000. The ranking scheme for MACS, exomePeak and MeTPeak is fold change. For MoAIMS, the ranking scheme of fold change(FC) and score are both used for comparison. Y-axis is the number of enriched regions with m6A miCLIP sites.

(a) Mouse WT_rep2

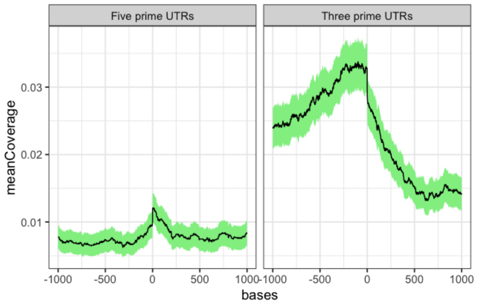

(b) Human shGFP_rep1

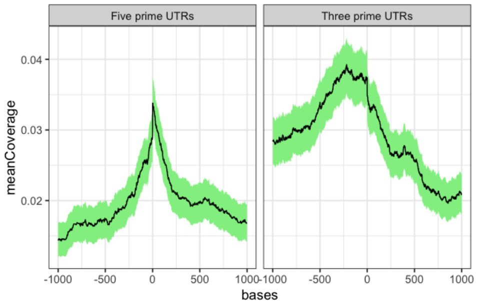

(c) Human shGFP_rep2

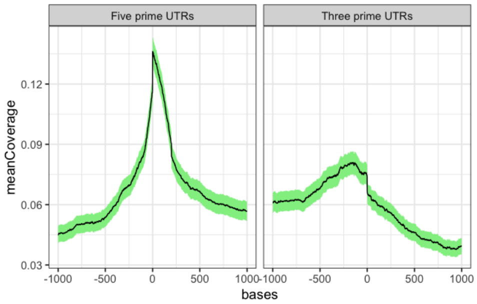

Figure S7 Position profile of m6A-enriched regions for the following datasets: WT_rep2 (wild-type mouse replicate 2), shGFP_rep1, and shGFP_rep2 (two replicates of human negative control). X-axis is the relative position coordinates and Y-axis is the mean coverage of the enriched regions. The plot is generated using RCAS[2].

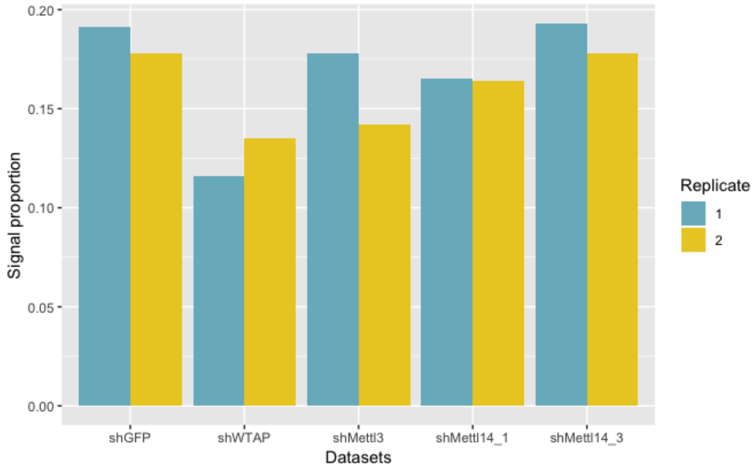

Figure S8 Signal proportion for m6A treatment experiments. X-axis represents five human MeRIP datasets of shGFP (negative control) and perturbation experiments including shWTAP, shMettl3, shMettl14_1, and shMettl14_3 with blue for replicate1 and yellow for replicate2. Y-axis represents the signal proportion.

Table S1: Information of MeRIP-Seq datasets

Name	Species	Type	Replicates
WT	Mouse	Wild	2
KO_Mettl3	Mouse	Treated	2
shGFP	Human	Negative control	2
shWTAP	Human	Treated	2
shMettl3	Human	Treated	2
shMettl14_1	Human	Treated	2
shMettl14_3	Human	Treated	2

Table S2: Consistency of enriched regions called by MoAIMS, MACS, exomePeak, and MeTPeak

Mouse WT_rep1	MoAIMS	MACS	exomePeak	MeTPeak
MoAIMS	100(11869)	72.9	64.8	71.0
MACS	81.3	100(14681)	69.2	80.9
exomePeak	96.4	96.6	100(19698)	91.5
MeTPeak	44.0	48.4	37.8	100(9049)

Mouse WT_rep2	MoAIMS	MACS	exomePeak	MeTPeak
MoAIMS	100(9411)	81.3	61.7	69.7
MACS	88.2	100(11161)	66.8	70.8
exomePeak	97.8	98.1	100(16190)	85.5
MeTPeak	61.9	61.9	49.7	100(10133)

WT_rep1 and WT_rep2 are two replicates of wild-type mouse datasets. Each cell is shown in percentage(%) and the number in bracket is the number of enriched regions.

Table S3: Length comparison of top-5000 enriched regions

Dataset	MoAIMS(score)	MoAIMS(FC)	MeTPeak	exomePeak	MACS
shGFP_rep1	400(400)	400(473)	399(458)	297(386)	244(297)
shGFP_rep2	400(549)	600(653)	300(365)	300(400)	221(271)

shGFP_rep1 andshGFP_rep2 are two replicates of the human negative control datasets. Each cell represents the median length, and the number in bracket is the mean length. The ranking scheme for MACS, exomePeak and MeTPeak is fold change. For MoAIMS, the ranking scheme of fold change(FC) and score are both used for comparison.

Table S4: Comparison of methods of counting reads in bins for pair-end sequencing

	MoAIMS		exomePeak		MeTPeak	
Dataset	Pair-end	First-read-in-pair	Pair-end	First-read-in-pair	Pair-end	First-read-in-pair
shGFP_rep1	14137	15319	24009	17573	14478	9213
shGFP_rep2	21603	24300	26741	18418	13610	7401

shGFP_rep1 and shGFP_rep2 are two human negative control datasets. Pair-end means using both reads in pair-end sequencing as input, while first-read-in-pair means using only the first read in pair-end sequencing. Each cell shows the number of enriched regions.

References

[1] Kuan, P.F., Chung, D., Pan, G., Thomson, J.A., Stewart, R., Keles, S.: A Statistical Framework for the Analysis of ChIP-Seq Data. J Am Stat Assoc 106(495), 891–903 (2011)

[2] Uyar, B., Yusuf, D., Wurmus, R., Rajewsky, N., Ohler, U., Akalin, A.: RCAS: an RNA centric annotation system for transcriptome-wide regions of interest. Nucleic Acids Res. 45(10), 91 (2017)
